# Targeted probiotic tabletting: A hybrid active learning and finite element modelling approach for process optimisation

**DOI:** 10.1016/j.ijpx.2025.100420

**Published:** 2025-10-17

**Authors:** Bide Wang, Xilu Wang, Oleksiy V. Klymenko, Jiawei Hu, Rachael Gibson, Andrew Middleton, Chuan-Yu Wu

**Affiliations:** a School of Chemistry and Chemical Engineering, University of Surrey, Guildford GU2 7XH, UK; b School of Computer Science and Electronic Engineering, University of Surrey, Guildford GU2 7XH, UK; c P&G Innovation Centre, Reading, Berkshire RG2 0QE, UK

**Keywords:** Active learning, Finite element method, Gaussian process regression, Probiotic, Tabletting

## Abstract

Tablets are an efficient dosage form for delivering probiotics. Prior studies have identified compression pressure, compression speed, and precompression pressure as critical process parameters determining probiotic survival during tabletting. However, due to the labour-intensive and time-consuming nature of experimental investigations, most previous studies focused on evaluating the impact of individual parameters in isolation. Consequently, the rapid and systematic identification of optimal process parameters to maximise probiotic survival remains a significant and unresolved challenge in pharmaceutical formulation research. To address this gap, an integrated approach combining active learning (AL) based Gaussian process regression (GPR) with finite element (FE) modelling was developed to systematically explore the compaction parameter space and identify optimal process conditions. All data utilised in AL were generated using an FE model that was specifically developed to predict viability of probiotics during tabletting. Remarkably, the integrated approach achieved high prediction performance after only 78 iterations, demonstrating a coefficient of determination (R^2^) of 0.96 across the entire design space for predicting probiotic survival rate during tabletting. Using the well-trained model, a global random sampling strategy combined with threshold filtering was employed to identify regions of the design space likely to yield near-optimal survival rates. Furthermore, the exploration of compression speed and precompression pressure at selected fixed main compression pressures enabled the generation of survival rate maps, providing insights into the interplay between probiotic survival rate and tablet mechanical performance. This study demonstrated the potential of hybrid data-driven and first-principles modelling approaches as a robust strategy for optimising probiotic tabletting processes and accelerating pharmaceutical development.

## Graphical abstract

**Figure:**
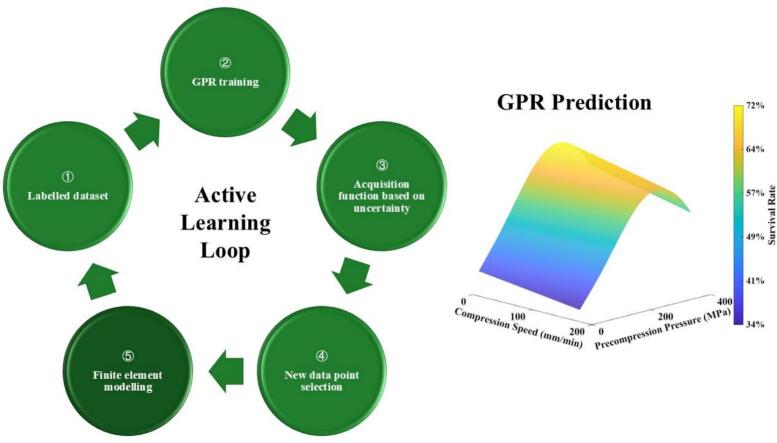

## 1 Introduction

Probiotics are live microorganisms that can provide health benefits to the host when consumed in sufficient amounts (Hill et al., 2014). These benefits include supporting immune function, enhancing digestive health, and promoting metabolism (Dong et al., 2012; Quigley, 2019; Russell et al., 2011). Probiotics can be delivered to the human body through various administration systems (Wang et al., 2024a; Xu et al., 2024), including functional foods, medical devices, and dietary supplements. Among these, tablets, as one of the common dosage forms of dietary supplements, have gained increasing popularity in recent years due to their stability and ease of production. However, numerous studies (Blair et al., 1991; Byl et al., 2019; Klayraung et al., 2009; Poulin et al., 2011; Vorländer et al., 2024) have demonstrated that conventional tabletting processes can significantly compromise probiotic viability, with substantial cell damage or mortality observed. These findings underscore the critical need for process optimisation to maintain therapeutic efficacy in the final product.

The study on the lethal mechanisms affecting viability of microorganisms during tabletting dates back to 1977, when Chesworth et al. (1977) proposed that the shear forces and localised heating generated during tabletting could damage cell structures and membranes, thereby reducing probiotic viability. Plumpton et al. (1986) further demonstrated that higher compression pressures could be lethal to microorganisms, based on their study on yeast. In a study on encapsulation of *Lactobacillus acidophilus*, Chan and Zhang (2002) observed similar trends and reported a near-linear reduction in microbial viability with increasing compression pressure during tabletting. The study further incorporated morphological analysis of probiotic cells pre- and post-compression, demonstrating substantial structural damage to cellular walls and membranes at compression pressures of 160 MPa. In addition, Muller et al. (2014) proposed a mathematical model to describe the relationship between compression pressure and probiotic viability. The model was based on the viability of two probiotic strains under different compression pressures, and provided a quantitative framework to predict how compression pressure affects microbial viability. Wang et al. (2024a) further validated the model's generalisation capability using data reported in the literature (Poulin et al., 2011; Vorländer et al., 2023a). Moreover, it was also shown that prolonging dwell time of the main compression pressure could reduce probiotic survival (Vorländer et al., 2023b).

In addition to compression pressure, whether other process parameters affect the viability of probiotics during tabletting was also examined. For instance, in a study on *Bacillus subtilis* tabletting, Ayorinde et al. (2013) observed that lower compression speeds resulted in higher death rate for this microorganism, which was attributed to increased mechanical damage and frictional heat experienced by microorganisms at lower compression speeds. However, this assertion is debatable, as Krok et al. (2016) in a thermomechanical analysis of tabletting showed that higher compression speeds lead to more irreversible work being converted into heat, thereby increasing internal temperatures. In addition to these findings, Vorländer et al. (2023b) systematically investigated the effect of consolidation time during tabletting of *Saccharomyces cerevisiae*. Consolidation times between 45 and 4990 ms (corresponding to compression speeds of approximately 40 to 0.4 mm/s) were evaluated. No significant correlation between compression speeds and probiotic survival was observed, and tablet porosity and tensile strength also remained essentially unaffected. This indicates that compression speed did not significantly affect microbial viability under the conditions studied. These contrasting findings clearly illustrate that conclusions regarding the role of compression speed on probiotic viability can differ substantially, which may be explained by strain-specific physiological responses and formulation-dependent mechanical/thermomechanical behaviour. Nevertheless, further systematic studies are required to clarify the role of compression speed in probiotic survival.

Precompression has recently attracted increasing attention due to its potential influence on probiotic viability. In a study on tabletting process optimisation for *Faecalibacterium prausnitzii* (Allouche et al., 2018), the application of a precompression pressure of 67 MPa prior to a main compression pressure of 201 MPa was reported to markedly increase survival compared to single compression at the same maximum pressure. However, the experimental variability in that study was relatively high, and the difference in survival rate was not statistically significant, indicating that further investigation is required. In one of our previous works (Wang et al., 2025a), the role of precompression pressure on the viability of *Lactobacillus gasseri* KS-13 was also explored. Finite element (FE) model predicted that the viability of *L. gasseri* KS-13 could be improved by approximately 0.2 log cycles when a precompression of 133 MPa was applied before a main compression of 374 MPa. Nevertheless, it must be acknowledged that the corresponding experimental data also showed considerable uncertainty. More recently, Vorländer et al. (2024) examined the influence of applying either 10 % of the main compression pressure as precompression or repeated compressions at the same pressure on the viability of *S. cerevisiae*. Their results indicated that a precompression with 10 % of main compression pressure had little influence, whereas repeated compressions at the same pressure reduced survival rate, which was attributed to decreased porosity. It should be noted, however, that their study investigated only two extreme cases of precompression. Whether these findings remain valid across a broader range of precompression pressures remains to be determined.

In summary, while existing studies have primarily examined compaction parameters in isolation, a critical knowledge gap persists regarding the synergistic effects of compression speed, precompression pressure, and main compression pressure—particularly in identifying optimal parameter combinations to maximise probiotic viability during tabletting. To date, no systematic investigation has comprehensively explored these interacting factors in probiotic tabletting processes. However, comprehensive experimental study of the effects of various combinations of compaction parameters on probiotic viability would be labour-intensive and time-consuming. Ideally, understanding probiotic viability across all combinations of parameters is essential to identify the optimal process conditions. Nonetheless, obtaining such extensive experimental data is impractical, even for formulations containing a single probiotic strain.

Nevertheless, recent advancements in machine learning (ML) offer a promising avenue to address this gap (Mäki-Lohiluoma et al., 2021; Wang et al., 2025b; Gormley, 2024; Hong et al., 2024; Sultan et al., 2024; Djuriš et al., 2021; Hayashi et al., 2021), and active learning (AL), a subset of ML, is particularly well-suited for developing ML models with small datasets (McCoubrey et al., 2021; Rakhimbekova et al., 2023; Patel et al., 2025). In the AL framework, a surrogate model is initially trained on a preliminary dataset and provides an estimate of uncertainty in its predictions throughout the parameter space. The AL framework then identifies additional points in the parameter space with highest uncertainty, where further data points are sampled to improve the accuracy of the surrogate model (Agnihotri and Batra, 2020). This approach is particularly advantageous when data are limited or costly to acquire, as it enables the model to learn more efficiently with fewer data points, as compared to uniform or random sampling of the parameter space, thereby accelerating the training process (Settles, 2009). For example, in a recent study on probiotic formulations, McCoubrey et al. (2022) utilised a small dataset comprising 6 probiotic-excipient interactions to train an AL-driven random forest classification model. With this model, they predicted the effects of 111 additional excipients on probiotic proliferation, achieving an accuracy of 75 %. This study highlights the potential application of AL in probiotic formulation development, while its role in optimising the tableting processes of probiotics warrants further exploration.

Therefore, the aim of the current study is to develop an AL-based surrogate model to identify optimal process conditions for *L. gasseri* KS-13, a widely utilised probiotic strain (Dennis-Wall et al., 2017; Spaiser et al., 2015), using a limited dataset initially generated using a validated FE model (Wang et al., 2025a). This integrated approach iteratively improves the model performance by identifying new data points with the highest prediction uncertainty for further FE simulation, thereby efficiently refining the model's predictive capability regarding probiotic survival rate. Additionally, the study aimed to use the refined model to identify optimal process conditions, ultimately providing a foundation for advancing process design strategies in probiotic tablet manufacturing.

## 2 Hybrid machine learning and finite element modelling

This section provides a detailed description of the AL-based Gaussian process regression (GPR) approach. In addition, the fundamental principles of the FE model used for data generation and the implementation of the alpha shape for identifying optimal process parameters are also introduced.

### 2.1 Overview of active learning

AL enhances training efficiency and surrogate model accuracy by selectively querying data points under specific process conditions, guided by predefined criteria (Settles, 2010). One of the most commonly used criteria for selecting data points is the reduction of maximum uncertainty of model predictions (Wang et al., 2024b), which is usually measured using the standard deviation.

**Fig. 1:**
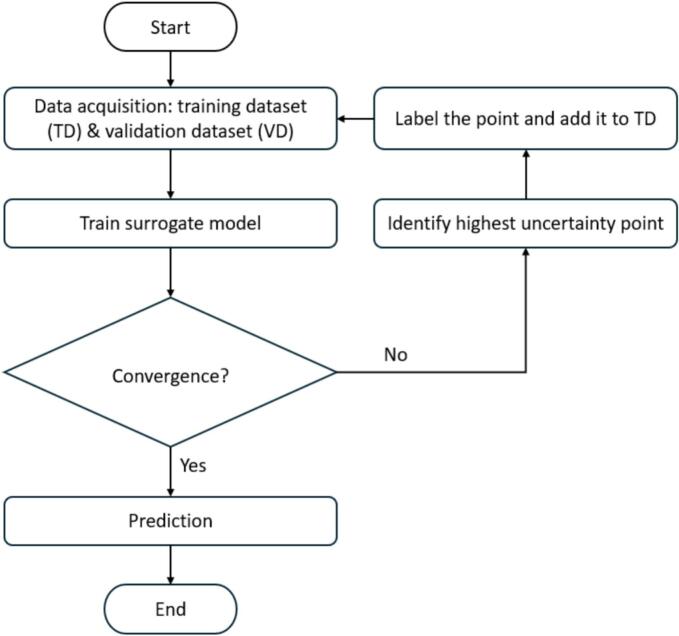
Flowchart of the AL approach.

In this study, the initial training dataset generated from FE model was first used to train the surrogate model. At each iteration of AL, the trained surrogate model was used to predict the standard deviation of probiotic survival rates, enabling the selection of a new data point with the highest uncertainty using a maximum standard deviation acquisition function (Wang et al., 2024b). The new data point was computed using the FE model and subsequently added to the training dataset to retrain the surrogate model, aiming to refine its posterior distribution. These iterations are repeated until the model reached its resource constraint. Fig. 1 illustrates the flowchart of the AL approach employed in this study. All model training and computations were performed on a computer with a 4-core CPU and 16 GB of RAM.

### 2.2 Data collection using the finite element model

#### 2.2.1 Finite element model

In this study, compression speed, precompression pressure, and main compression pressure were selected as the three input parameters in ML, while the corresponding probiotic survival rate served as the output. Each unique combination of input parameters and its associated survival rate prediction constituted a single data point. The survival rate values were computed using the FE model developed in our earlier work (Wang et al., 2025a), where full details on model parameterisation and implementation are available. To maintain the focus and coherence of the current study, only a brief overview of this model is provided here. Developed using the commercial software Abaqus/Standard 2023 (Dassault Systèmes, France), the FE model captures three fundamental mechanisms involved in the probiotic tabletting process:

i) Mechanical behaviour. The Drucker Prager Cap (DPC) model is used to describe the mechanical behaviour of powder during compaction, and comprises three surfaces (Seville and Wu, 2016):

The shear failure surface, $F_{s}$:

(1) $$\mathrm{Fs}\;\left(p,q\right)=q-p\;\mathrm{tan\beta}-d=0$$

The cap surface, $F_{c}$:

(2) $$F_{c}\left(p,q\right)=\sqrt{{\left(p-p_{a}\right)}^{2}+{\left(\left(\hat{R}q\right)/\left(1+\alpha -\alpha /\mathrm{cos\beta}\right)\right)}^{2}}-\hat{R}\left(d+p_{a}\mathrm{tan\beta}\right)=0$$

The transition surface, $F_{T}$:

(3) $$F_{T}\left(p,q\right)=\sqrt{{\left(p-p_{a}\right)}^{2}+{\left[q-\left(1-\alpha /\mathrm{cos\beta}\right)\left(d+p_{a}\mathrm{tan\beta}\right)\right]}^{2}}-\alpha \left(d+p_{a}\mathrm{tan\beta}\right)=0$$

where p and q are the hydrostatic stress and the von Mises equivalent stress. d and β are the cohesion and the angle of friction, α is a parameter that defines the shape of the transition surface, $p_{a}\;$ is the parameter characterising the hardening/softening behaviour of the materials, and is function of $\varepsilon_{v}$, and $\hat{R}$ is the eccentricity of the curve.

ii) Thermomechanical response. An energy balance equation is introduced to describe the thermomechanical behaviour by considering friction between the powder particles and powder/tooling, as well as the plastic deformation of the particles during compaction. It can be expressed as (Krok et al., 2016; Krok and Wu, 2017):

(4) $$\rho C_{p}\frac{\mathrm{dT}}{\mathrm{dt}}=\nabla \left(k\nabla T\right)+{\dot{q}}_{p}+{\dot{q}}_{f}$$

where ρ is the local density, $C_{p}$ and k denote the specific heat capacity and thermal conductivity, respectively. ∇T is the temperature gradient, ${\dot{q}}_{p}$ and ${\dot{q}}_{f}$ are the rate of plastic heat generation and rate of frictional heat generation.

iii) Thermal tolerance. A thermal tolerance model is implemented to determine the viability based on the temperature distribution $T\left(x,y,z\right)$ at each location $\left(x,y,z\right)$ during compaction. The thermal tolerance model enables the overall probiotic viability to be calculated as follows (Wang et al., 2025a):

(5) $$\bar{S}=\frac{1}{V}∭S\left(T\left(x,y,z\right)\right)\mathrm{dxdydz}$$

where V is the volume of the tablet, and $S\left(T\right)$ is the function of the viability of the probiotics after a transient exposure to temperature T.

#### 2.2.2 Data space

Since tablets must be robust enough to withstand storage and transportation (Osei-Yeboah and Sun, 2015), yet not so strong as to hinder disintegration in the body (Momeni et al., 2024), this study only considered the main compression pressure ranging between 50 MPa and 400 MPa. For precompression pressure, the constraint is that it must not exceed the main compression pressure. A broad compression speed range of 0 to 5000 mm/min was explored to accommodate both laboratory-scale and operating conditions of modern industrial presses, enabling a comprehensive assessment of process feasibility.

The survival rate was selected as the output metric, as it offers a more intuitive and standardised representation of probiotic viability by normalising results to a fixed reference. This normalisation mitigates bias arising from initial cell count variability while enabling more robust cross-condition comparisons. Furthermore, expressing survival rate as a percentage enhances both interpretability and the visual clarity of reported outcomes.

The initial training dataset consisted of 30 data points selected using Sobol sampling—a low-discrepancy, quasi-random method designed to ensure uniform and well-distributed coverage of the input parameter space (Burhenne et al., 2011). This sampling strategy enhances model initialisation, supports accurate uncertainty estimation, and accelerates learning efficiency during the early stages of model development. To ensure a fair and comprehensive evaluation of the model's generalisation performance, the validation dataset was generated using the same Sobol sampling approach. This consistent methodology minimises evaluation bias, promotes stability, and maintains uniformity across the sampled design space.

### 2.3 Surrogate model implementation

The Statistics and Machine Learning Toolbox in MATLAB was utilised to develop all the surrogate models in this study. The GPR model was specifically chosen in this study due to its ability to handle small datasets and capture complex nonlinear relationships. Additionally, it provides uncertainty quantification, which can seamlessly integrate with AL. A GPR model can be specified by a mean function and a kernel function (Wang and Jin, 2023):

(6) $$f\left(x\right)∼\mathrm{GP}\left(m\left(x\right),k\left(x,x^{\prime }\right)\right)$$

where $f\left(x\right)$ is the function to be learned, $m\left(x\right)$ is the mean function (often assumed to be zero), and $k\left(x,x^{\prime }\right)$ is the kernel function that defines the covariance between any two points x and $x^{\prime }$.

The kernel function is a key component of the GPR model, which serves as a measure of similarity between data points (Pan et al., 2021). The choice of kernel function directly affects the performance of the GPR model, including both fitting capability and smoothness of the model prediction. In this study, the squared exponential (SE) kernel was used due to its ability to provide smooth and continuous function approximations while effectively capturing complex, nonlinear relationships within the data space. The SE kernel can be expressed as (Wang et al., 2023):

(7) $$k_{\mathrm{SE}}\left(x,x^{\prime }\right)=\sigma^{2}\exp\left(-\frac{{\left(x-x^{\prime }\right)}^{2}}{2l^{2}}\right)$$

where l is the length-scale parameter that determines how rapidly the function changes. $\sigma^{2}$ denotes the variance parameter that controls the overall scale of function variability. Both parameters were determined using Bayesian optimisation (BO) with the *fitrgp* function in MATLAB. Each BO run involved 30 iterations using the expected improvement plus (EI+) method, an enhanced expected improvement (EI) acquisition function (Bull, 2011) that can avoid a local objective function minimum, to identify the optimal parameter set and ensure efficient model tuning. Two commonly used evaluation metrics in regression problems, the coefficient of determination (R^2^) and root mean square error (RMSE), were employed in the assessment.

### 2.4 Optimal process identification

To identify the potential parameter combinations that maximise the probiotic survival rate, the trained GPR model was used to predict survival rates across the design space. A total of 5000 candidate points were randomly sampled within the feasible ranges of compression speed, precompression pressure, and main compression pressure. Points satisfying the practical constraint that precompression pressure was less than the main compression pressure were retained.

To account for prediction uncertainty and identify a high-survival region rather than a single point, candidate points with predicted mean values within 99 % of the maximum were selected as the potential optimal region. The resulting points were then visualised in three-dimensional design space using an alphaShape (Edelsbrunner and Mücke, 1994), highlighting the feasible high-survival domain under the process constraints.

## 3 Experimental validation

A series of tabletting experiments and survival rate tests were conducted to validate and evaluate the hybrid approach discussed in Section 2.

### 3.1 Materials

The formulation for tabletting was supplied by Procter & Gamble (Austria). The main ingredient of the formulation is freeze-dried *L. gasseri* KS-13. De Man–Rogosa–Sharpe (MRS) agar and phosphate buffered saline (PBS) were sourced from VWR International Ltd. (UK) and Fisher Scientific (UK), respectively. All other solvents and chemicals were of laboratory grade.

### 3.2 Tablet preparation

Probiotic tablets with a fixed mass of 130 mg were produced using a Gamlen D1000 powder compaction analyser (Gamlen, UK) equipped with a 6 mm diameter die. The compression speed was set at 180 mm/min, and the main compression pressure was maintained at 208 MPa. The survival rates of probiotic tablets compressed under varying precompression pressures (0–208 MPa) were compared against the GPR model predictions. To further verify the accuracy of the identified optimal compaction design space, representative points were selected, and tablets were produced according to the corresponding parameter settings. The survival rates of these tablets were then measured and compared with other experimental data. For each experimental condition, six replicate tablets were prepared to ensure reproducibility. All process parameter combinations are listed in Table 1. As a key mechanical property of tablets, porosity was also measured in this study. However, since this property is not the main focus in this study, the corresponding data are provided in the Supplementary materials for reference.

**Table 1:** Parameter settings for experimental tabletting validation.

Case	Compression speed (mm/min)	Precompression pressure (MPa)	Main compression pressure (MPa)
1	180	0	208
2	180	10	208
3	180	62	208
4	180	121	208
5	180	173	208
6 (Optimal)	160	37	109
7 (Optimal)	28	29	147

### 3.3 Survival rate determination

After compaction, the tablet was immersed in 50 ml of PBS for 30 min and then vortexed for 5 min. The resulting suspension was serially diluted in 10 ml PBS to the desired cell concentration, and two aliquots of each dilution were inoculated onto MRS agar plates. The plates were incubated under microaerophilic conditions at 37 °C for up to 72 h. All tests were performed in duplicate to ensure the reliability and consistency of the results. The probiotic viability per tablet was then determined by counting the colony-forming unit (CFU) on the plates:

(8) $$S=\left(N_{P}*V_{I}\right)/\left(r*V_{F}\right)$$

where $N_{P}$ represents the CFU count on the plate, $V_{I}$ is the initial suspension volume, r denotes the dilution factor, and $V_{F}$ is the volume of the final suspension plated. And the survival rate (SR) of the probiotics can then be calculated as:

(9) $$\mathrm{SR}=S/S_{b}\times 100\%$$

where $S_{b}$ denotes the probiotic viability in the as-received formulation used for producing one tablet, which was determined to be 3.9 × 10^7^ CFU/tablet.

## 4 Results and discussion

This section first presents an overview of the dataset used for GPR model training, describing its structure and key features. Next, it outlines the model development process before providing an in-depth analysis. The section concludes with an evaluation and discussion of predicted optimal compaction conditions.

### 4.1 Dataset overview

**Fig. 2:**
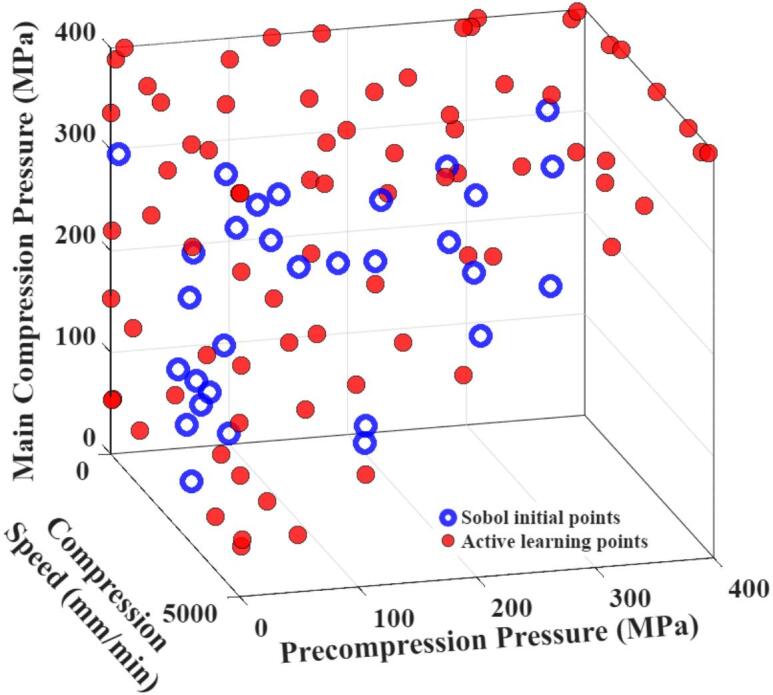
Distribution of the training data (including initial Sobol sampling points and AL points) in the defined input parameter space.

Fig. 2 provides a comprehensive visualisation of the distribution of training data points in the input parameter space. The initial training dataset (blue open circles) consists of 30 data points generated via quasi-random Sobol sampling, while the AL dataset (red solid circles) contains an additional 78 AL points.

A straightforward observation is that no training points appear in the lower-right region of the figure. This is because, as explained in Section 2.2.2, the precompression pressure was constrained to be lower than the main compression pressure, which inherently excluded this region from the sampling domain. In addition, it is evident that AL tends to allocate more sampling points near the boundaries of the parameter space for all variables, reflecting the higher prediction uncertainty in these regions. Consequently, the AL algorithm prioritises querying boundary areas to effectively reduce such uncertainty. Furthermore, the initial GPR model was trained on the Sobol points (blue open circles), and during the subsequent AL process, the queried points (red solid circles) were mostly located away from these initial samples. This behaviour arises because, once the model has been trained on the Sobol points, it can already provide low-uncertainty and accurate predictions in their vicinity. Since the essence of AL is to query points with the highest prediction uncertainty, the AL points naturally avoid regions surrounding the Sobol points. Finally, it can also be observed that certain regions were sampled repeatedly. This is because the model exhibited high prediction uncertainty in those regions, leading the AL algorithm to preferentially select multiple points within these regions to gradually reduce the uncertainty.

### 4.2 GPR model development

**Fig. 3:**
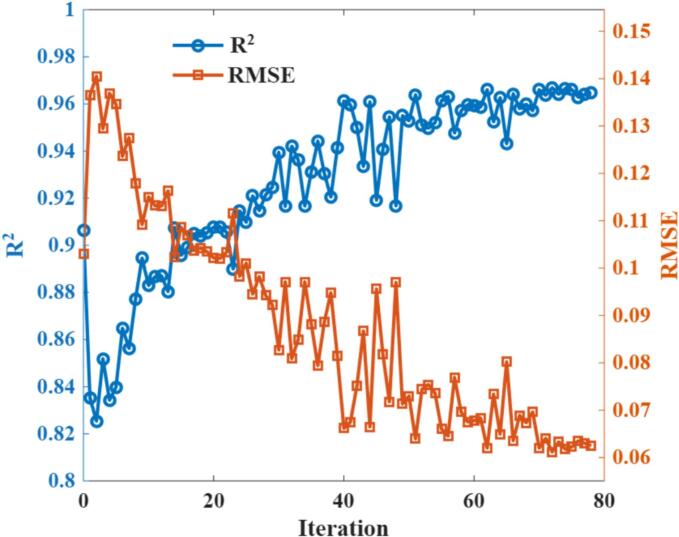
Performance of GPR models across different iterations.

Fig. 3 illustrates the trends of RMSE and R^2^ across different iterations of the GPR models during AL. Evidently, the model performance exhibited a marked decline when transitioning from the initial 30 Sobol points to the first AL iteration. This phenomenon typically occurs when the model is first exposed to newly queried samples located in regions of high uncertainty, where the information has not yet been sufficiently learned, resulting in a temporary increase in prediction error. As the number of AL iterations increased, the model progressively assimilated this new information, and its performance steadily improved until convergence. In this study, a total of 78 iterations were carried out, yielding an R^2^ of 96 % and reducing the RMSE to approximately 0.06, representing a substantial improvement compared to the initial model.

### 4.3 GPR model validation

In this section, the AL-based GPR model developed in this study was used to predict probiotic survival rate for various main compression pressures (with experimental data reported in (Wang et al., 2025a)) and precompression pressures. These predictions were then systematically compared with corresponding experimental results to evaluate the model's accuracy and reliability in capturing the complex relationships between compaction parameters and probiotic survival rate.

**Fig. 4:**
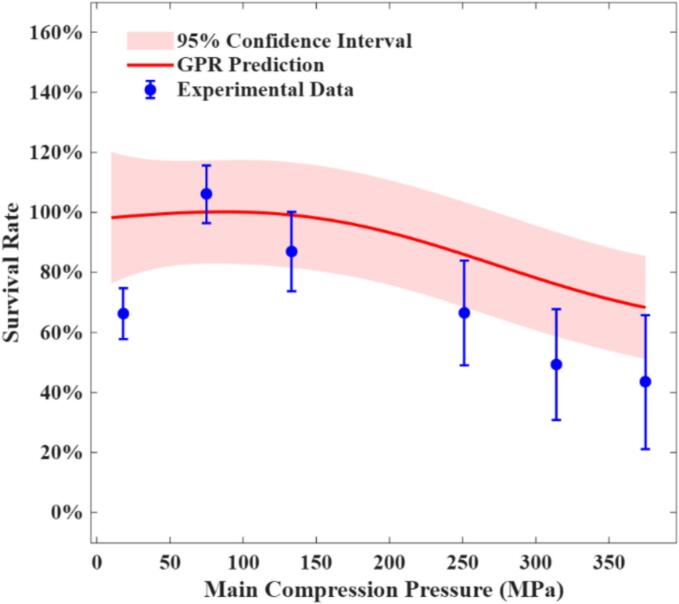
Comparison of experimental survival rate and GPR predictions across varying main compression pressures under a fixed main compression speed (180 mm/min). Experimental data (*n* = 3) were obtained from our previous study (Wang et al., 2025a).

The probiotic survival rate under various main compression pressures at a fixed compression speed was experimentally measured and compared with the predictions generated by the GPR model, as shown in Fig. 4. At low compression pressures, survival rates exhibited a considerable increase, which can possibly be attributed to two possible mechanisms: activation of dormant cells (Plumpton et al., 1986) and membrane repair of sub-lethally damaged cells during rehydration (Vorländer et al., 2023a). Overall, however, the GPR-predicted values are in good agreement with the experimental results, accurately capturing the overall trend of decreasing survival rate with increasing compression pressure. This trend is well-supported by previous observations reported in the literature (Vorländer et al., 2023a; Allouche et al., 2018; Byl et al., 2020), which have consistently demonstrated that high compression pressures result in increased microbial loss within tablets. These results collectively highlight that minimising main compression pressure is a critical strategy for preserving probiotic survival rate during the tabletting process.

**Fig. 5:**
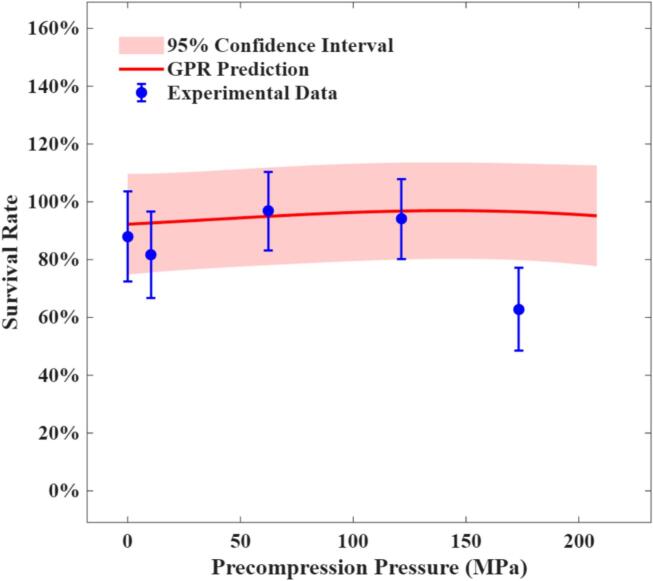
Comparison of experimental survival rate (*n* = 6) and GPR predictions across varying precompression pressures under a fixed main compression pressure (208 MPa) and compression speed (180 mm/min).

The relationship between precompression pressure and probiotic survival rate was further examined at a fixed compression speed of 180 mm/min and a main compression pressure of 208 MPa, as shown in Fig. 5. Precompression pressure led to a modest and gradual increase in survival rate, although the overall improvement was limited. At higher precompression pressures, the experimental data showed a decline in probiotic survival. This increased probiotic death can be attributed to the probiotics effectively undergoing a "double compression" process. Unlike lower precompression pressures, which primarily contribute to gentle heat dissipation and potentially protective effects (Wang et al., 2025a), higher precompression subjects the probiotics to repeated mechanical stress and associated heat generation, resulting in repeated heat shock. A similar phenomenon was reported by Vorländer et al. (2024), who attributed the reduced probiotic survival under repeated compression to a decrease in tablet porosity, further exacerbating probiotic death. It should also be noted that a noticeable deviation was observed between the GPR model predictions and experimental results at higher precompression pressures. This discrepancy arises because the GPR model primarily improves global uncertainty, and capturing variations in specific regions requires additional iterations to be fully resolved.

In general, the GPR model demonstrates a strong ability to accurately capture the variation in probiotic survival rate across different compaction conditions. Although some discrepancies exist between the predicted and experimental results, these deviations are expected, given that the model's RMSE remains around 0.06 after 78 AL iterations. Nonetheless, the optimal conditions identified by experiments fall within the range predicted by the GPR model, with only minor differences. This consistency confirms that the model is sufficiently accurate in identifying and predicting optimal compaction conditions in practice.

### 4.4 Design space

**Fig. 6:**
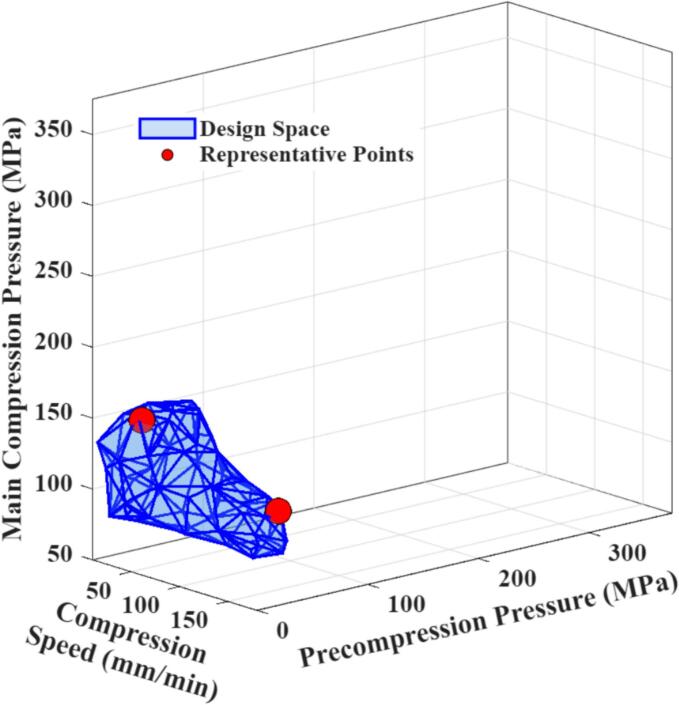
Alpha Shape representation of optimal regions for probiotic survival within the given compaction parameter space.

An exploration of the compaction design space was conducted within the operational limits of the Gamlen D1000 compaction analyser available in our laboratory, which allows for compression speeds between 10 and 180 mm/min and pressures between 0 and 374 MPa (with a minimum main compression of 50 MPa). Fig. 6 illustrates the potential optimal regions for probiotic survival in three-dimensional compaction parameter space, as predicted by the trained GPR model.

Within this compaction parameter space, the predicted optimal conditions for probiotic survival correspond to a main compression pressure of approximately 70–150 MPa and a precompression pressure ranging from 0 to 75 MPa, while compression speed has a relatively minor impact. Overall, these results are reasonable: lower main compression pressures preserve higher probiotic survival, and as shown in Fig. 4, survival exhibits a relatively broad optimal region within the low main pressure range.

To further verify the reliability of the identified optimal design space, two representative points were chosen, corresponding to the conditions with the higher compression speed and higher main compression pressure within this region. The experimental results for probiotic survival rates under these conditions are summarised in Table 2. Notably, probiotic survival remained virtually unchanged in these cases, confirming that this region indeed represents the optimal compression domain.

**Table 2:** Survival rates of representative points selected from the design space (*n* = 6).

Case	GPR survival rate (%, mean ± SD)	Experimental survival rate (%, mean ± SD)
6 (Optimal)	100 ± 9.6	100.4 ± 12.8
7 (Optimal)	100 ± 9.8	99.5 ± 13.8

Considering industrial manufacturing requirements, probiotic survival is not the sole critical attribute of probiotic tablets, as sufficient mechanical strength is also required to ensure product robustness. Since main compression pressure strongly influences tablet porosity (Vorländer et al., 2023a; Vorländer et al., 2023b), which in turn governs mechanical strength, the variation of probiotic survival was further investigated under specific fixed main compression pressures. For this analysis, compression speed was restricted to 4000–5000 mm/min, representing the upper range of the modelled data, which approaches typical industrial production speeds. Precompression pressure was limited to less than 50 MPa. Although evidence (Wang et al., 2025a) suggests that precompression may reduce the thermal stress experienced by probiotics during compaction, excessive precompression can trap air within the tablet and cause fracture or cracking during decompression (Mazel and Tchoreloff, 2022; Vreeman and Sun, 2024). Therefore, a conservative limit was applied. It should be noted that the parameter ranges considered here serve primarily as a reference and can be further adjusted depending on practical manufacturing requirements.

**Fig. 7:**
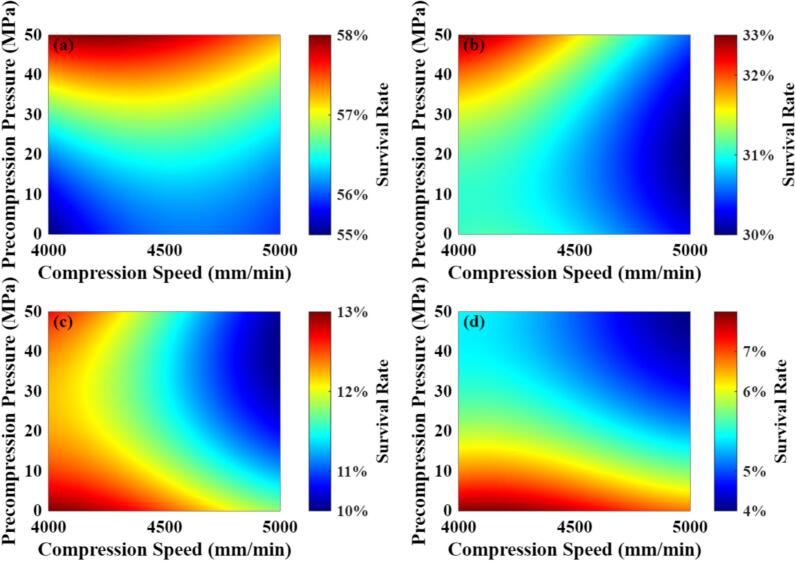
Probiotic survival rate across compression speeds (4000–5000 mm/min) and precompression pressures (0–50 MPa) under different main compression pressures: 250 MPa (a), 300 MPa (b), 350 MPa (c), and 400 MPa (d).

Fig. 7 illustrates the distribution of probiotic survival across compression speed and precompression pressure under varying main compression pressures. Overall, survival gradually decreases as the main compression pressure rises from 250 MPa to 400 MPa, reaching a minimum of approximately 4 % at 400 MPa. At 250 MPa, compression speed has a limited effect on survival, while a proper precompression pressure (∼50 MPa) can enhance survival by around 3 %, as precompression helps mitigate transient heat accumulation during main compression and reduces thermal damage to the probiotics (Wang et al., 2025a). At 300 MPa, appropriate precompression combined with lower compression speed significantly improves the survival rate, indicating that at medium-to-high pressures, higher compression speeds lead to increased frictional heat generation and reduce probiotic survival (Wang et al., 2025c). At 350 MPa, the influence of precompression begins to diminish, while compression speed starts to dominate the survival outcome. At 400 MPa, omitting precompression appears sufficient to achieve relatively high survival, and compression speed has almost no effect. This is because, in the FE simulations, the tooling acts as an efficient heat sink, limiting temperature accumulation to the tablet core and resulting in relatively low temperature changes (corresponding to minimal change in probiotic survival) at the tablet edges (Wang et al., 2025c). In actual production, however, continuous operation of the tablet press generates additional heat from friction and particle deformation, which dissipates into both the environment and tooling (Wang et al., 2025a). Under these conditions, the entire tablet experiences thermal stress, and probiotic survival in practice could therefore be lower than predicted by the FE model.

It is hence shown that the main compression pressure plays a dominant role in determining probiotic survival. Once the main compression pressure is fixed, adjusting compression speed and precompression within acceptable ranges can lead to modest improvements in survival rate.

Overall, this study aims to assist process engineers in rapidly identifying suitable compaction parameters during the early phase of process development, following initial formulation design. Therefore, formulation design aspects, such as probiotic strain selection and granule size, were not within the scope of this work. However, these factors are known to influence microbial survival during tabletting. Plumpton et al. (1986) demonstrated that different microorganisms, such as *S. cerevisiae* and *Aspergillus niger*, exhibit distinct responses to compaction pressure, reflecting strain-dependent tolerance. They also showed that the relative size of microorganisms and excipient particles is critical, with smaller cells embedded in larger carrier particles experiencing less stress and achieving higher survival. In addition, the influence of tablet geometry and punch shape, although not considered in the present study, has been shown to significantly alter the thermomechanical environment during compaction. FE modelling by Krok et al. (2016) demonstrated that convex punches generate higher deformation work and lead to greater heat accumulation compared with flat punches, thereby producing higher maximum tablet temperatures. Since thermal stress is also one of the major contributors to probiotic inactivation, these findings suggest that tablet geometry and punch design are also likely to affect probiotic survival. Although such aspects remain outside the present scope, they will undoubtedly become important considerations in our future investigations. Nevertheless, the AL-driven GPR model proposed herein has been thoroughly validated, demonstrating its effectiveness. This framework could be further extended to incorporate formulation– and geometry–related factors, thus providing a more holistic optimisation tool for probiotic tablet manufacturing. This study therefore establishes a solid foundation for future hybrid model-based and data-driven optimisation strategies.

## 5 Conclusions

A hybrid model integrating an AL-based Gaussian process regression model with FE modelling was developed to predict the survival rate of probiotics during tabletting. The FE model of probiotic survival was previously developed and calibrated against a relatively small set of experimental observations. The GPR model was initially trained on a small dataset of simulated probiotic survival rates under a range of tabletting conditions (compression speed, precompression pressure and main compression pressure). Through the AL framework, additional data points were iteratively selected for further FE simulations of probiotic viability to refine the GPR model by reducing the maximum uncertainty of its predictions at each iteration. The final model achieved an R^2^ value of 96 %, accurately capturing the probiotic survival trends under varying process conditions.

The resulting surrogate model allows the quantitative exploration of probiotic viability as a function of the tabletting parameters without the need for further experimentation. Using this model, optimal compaction parameters within the given parameter space were identified, illustrating the capability of this approach to efficiently optimise pharmaceutical process conditions for a specific formulation. This study underscores the advantages of hybridising data-driven and first-principles modelling approaches, offering a powerful and practical strategy for process optimisation and formulation design in pharmaceutical manufacturing.

## CRediT authorship contribution statement

**Bide Wang:** Writing – review & editing, Writing – original draft, Validation, Methodology, Formal analysis, Data curation, Conceptualization. **Xilu Wang:** Writing – review & editing, Supervision, Methodology, Formal analysis, Conceptualization. **Oleksiy V. Klymenko:** Writing – review & editing, Supervision, Methodology, Formal analysis, Conceptualization. **Jiawei Hu:** Methodology, Formal analysis. **Rachael Gibson:** Writing – review & editing, Supervision, Conceptualization. **Andrew Middleton:** Writing – review & editing, Supervision, Conceptualization. **Chuan-Yu Wu:** Writing – review & editing, Supervision, Project administration, Investigation, Funding acquisition, Conceptualization.

## Declaration of competing interest

The authors declare that they have no known competing financial interests or personal relationships that could have appeared to influence the work reported in this paper.

## Data Availability

Data will be made available on request.

## Appendix A Supplementary data

Supplementary material: Tablet porosity calculation Image 1
