# Supplementary material for: Targeted probiotic tabletting: A hybrid active learning and finite element modelling approach for process optimisation
Source: Int J Pharm X. 2025 Oct 17;10:100420. doi: 10.1016/j.ijpx.2025.100420 (PMC12590431; doi:10.1016/j.ijpx.2025.100420)
Supplement: Supplementary file 1 — Supplementary material: Tablet porosity calculation [file mmc1.docx]

**Supplementary data**

The following tablet porosity ($\varepsilon$) was calculated via [1]:

$\varepsilon=1-\frac{m_{t}}{\rho_{True}*\pi*h_{t}*{r_{t}}^{2}}$ (1)

where $m_{t}$, $h_{t}$, and $r_{t}$ represent the mass, thickness, and radius of the tablet produced.


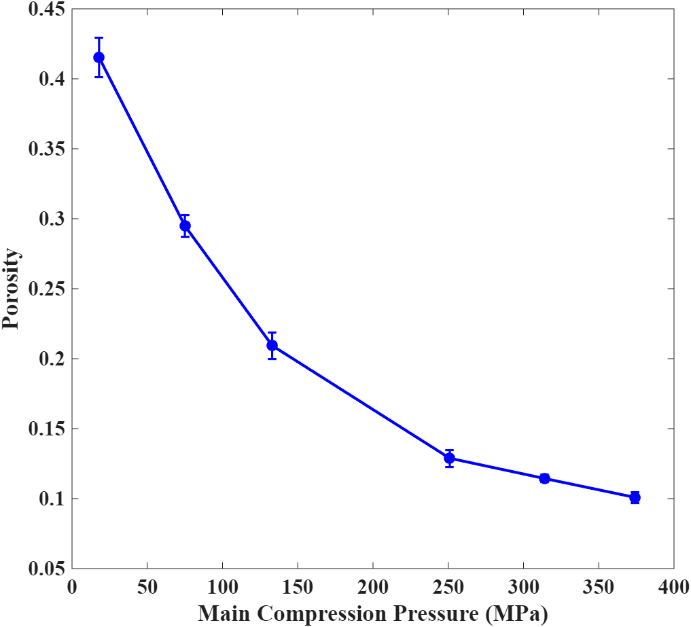


Supplementary Figure 1. Experimental tablet porosity across varying main compression pressures under a fixed main compression speed (180 mm/min).


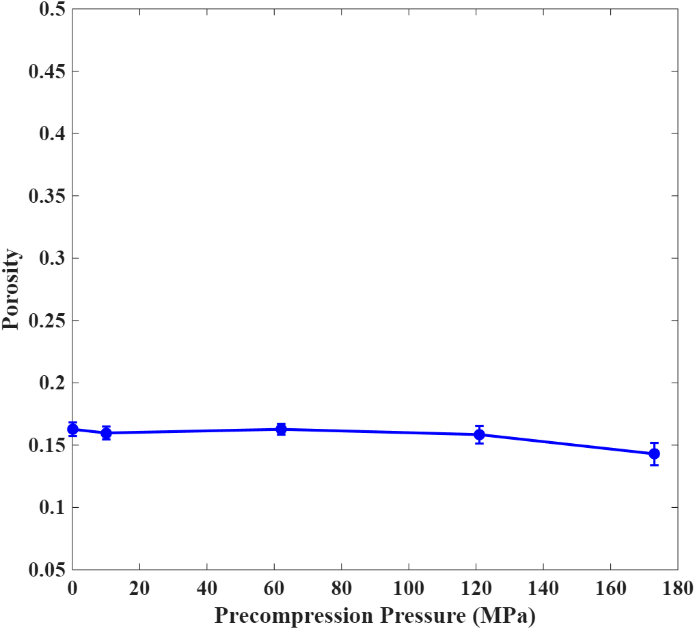


Supplementary Figure 2. Experimental tablet porosity across varying precompression pressures under a fixed main compression pressure (208 MPa) and compression speed (180 mm/min).

**Reference**

[1] C.C. Sun, Microstructure of tablet—pharmaceutical significance, assessment, and engineering, Pharmaceutical research, 34 (2017) 918-928.
